# Causes of Death in a Contemporary Cohort of Patients with Invasive Aspergillosis

**DOI:** 10.1371/journal.pone.0120370

**Published:** 2015-03-24

**Authors:** Carolina Garcia-Vidal, Maddalena Peghin, Carlos Cervera, Carlota Gudiol, Isabel Ruiz-Camps, Asunción Moreno, Cristina Royo-Cebrecos, Eva Roselló, Jordi Puig de la Bellacasa, Josefina Ayats, Jordi Carratalà

**Affiliations:** 1 Department of Infectious Diseases, Hospital Universitari de Bellvitge, IDIBELL (Institut d´Investigació Biomèdica de Bellvitge), Universitat de Barcelona, Barcelona, Spain; 2 REIPI (Spanish Network for Research in Infectious Diseases), Barcelona, Spain; 3 Hospital Universitari de la Vall d’Hebron, Barcelona, Spain; 4 Hospital Clínic i Provincial de Barcelona, Barcelona, Spain; Fred Hutchinson Cancer Center, UNITED STATES

## Abstract

Information regarding the processes leading to death in patients with invasive aspergillosis (IA) is lacking. We sought to determine the causes of death in these patients, the role that IA played in the cause, and the timing of death. The factors associated with IA-related mortality are also analyzed. We conducted a multicenter study (2008-2011) of cases of proven and probable IA. The causes of death and whether mortality was judged to be IA-related or IA-unrelated were determined by consensus using a six-member review panel. A multivariate analysis was performed to determine risk factors for IA-related death. Of 152 patients with IA, 92 (60.5%) died. Mortality was judged to be IA-related in 62 cases and IA-unrelated in 30. The most common cause of IA-related death was respiratory failure (50/62 patients), caused primarily by *Aspergillus* infection, although also by concomitant infections or severe comorbidities. Progression of underlying disease and bacteremic shock were the most frequent causes of IA-unrelated death. IA-related mortality accounted for 98% and 87% of deaths within the first 14 and 21 days, respectively. Liver disease (HR 4.54; 95% CI, 1.69-12.23) was independently associated with IA-related mortality, whereas voriconazole treatment was associated with reduced risk of death (HR 0.43; 95% CI, 0.20-0.93). In conclusion, better management of lung injury after IA diagnosis is the main challenge for physicians to improve IA outcomes. There are significant differences in causes and timing between IA-related and IA–unrelated mortality and these should be considered in future research to assess the quality of IA care.

## Introduction

Invasive aspergillosis (IA) is a leading cause of infection-related death in immunocompromised patients [1–4]. Recent data suggest that the outcomes of this infection appear to be improving compared with observations in the 1990s, this being due to advances in diagnosis and the introduction of new antifungal agents [5–8].

However, knowledge regarding the cause of death in patients with IA remains scarce for a number of reasons. First, patients with IA are complex hosts with severe underlying diseases, aggressive treatments, coexisting infections, and/or treatment complications. Second, studies focusing specifically on the processes leading to death in these patients are lacking. Third, a multidisciplinary approach involving a large number of physicians is common in the management of these patients, and the final cause of death reported on the death certificate may depend on the experience of the signing physician. And fourth, there is no clear consensus about how to define the cause of a patient’s death or the role that IA may have played in this event. This knowledge gap limits our understanding of optimal treatment strategies. It is not clear, therefore, whether IA-related mortality is caused by factors that could be modifiable through medical intervention.

Previous studies of IA prognosis have focused mainly on overall mortality, and they have assessed risk factors for all-cause mortality [7]. Moreover, definitions of IA-related mortality are vague [5, 6, 8]. A further aspect requiring elucidation is whether causes of death and factors associated with mortality within the first few days differ from those associated with mortality occurring later.

The present study sought 1) to identify the immediate causes of death in a contemporary cohort of hospitalized patients with IA, 2) to determine the role that IA played in the cause of death, and 3) to analyze the timing of death and risk factors associated with IA-related mortality in this cohort of patients.

## Materials and Methods

### Setting, patients, and study design

We conducted a retrospective multicenter study of all adults diagnosed with IA between 1 January 2008 and 31 December 2011 at three tertiary teaching hospitals in Barcelona, Spain. Patients who developed IA were identified by review of clinical records, of microbiology and pathology records, and of the diagnostic codes recorded on hospital discharge. The following information was carefully collected from medical records: demographic characteristics, underlying disease, use of immunosuppressive treatment, neutropenia, clinical features, diagnostic tools, infecting *Aspergillus* species, antifungal and adjunctive treatment, and outcomes. Informed consent was waived by the Clinical Research Ethics Committee because no intervention was involved and no patient identifying information was included. The study was approved by the Ethical Committee of the Hospital Universitari de Bellvitge.

### Definitions

We included only patients with proven and probable IA according to the definitions published by the European Organization for Research and Treatment of Cancer/National Institute of Allergy and Infectious Diseases Mycosis Study Group (EORTC/MSG) [9]. Neutropenia was defined as an absolute neutrophil count of <500/mm^3^. Disseminated IA was defined as evidence of infection in at least two noncontiguous sites or isolated CNS infection. The day of IA diagnosis was the day on which the first positive test was performed. For patients whose diagnosis was obtained from postmortem examination, the day of death was considered to be the day of diagnosis.

### Assessment of mortality and the cause of death

Mortality was assessed at 90 days from day of diagnosis (overall mortality). Cause of death and the role of IA in causing death were reviewed by members of clinical review panel. This panel was composed by 6 investigators. Data from each study’s patient were independently reviewed by three of these investigators. Results were based on full consensus among the investigators. The investigators were blind to the patient’s day of death. Five members of the clinical review panel were infectious disease specialists and one was an advanced fellow in infectious diseases. All reviewers had extensive clinical experience dealing with patients with IA. The reviewers were asked to assign the immediate causes of death based on World Health Organization criteria [10], and to assess the role that IA played in the patient’s death.

The immediate cause of death was defined as the disease process, injury, or complication immediately preceding death. IA was considered the cause of death when the immediate cause of death was due to this infection. IA was judged to have played a major role if death would not have occurred had the patient not had IA, even though another condition was present that also contributed to death. IA was defined as playing a minor role if IA was not essential in explaining the patient’s death but did play some role in the event. Mortality was classified as IA-related if IA was the cause of death or if it played a major role in the patient’s death. Mortality was defined as IA-unrelated if IA played a minor role or had no role in the patient’s death.

### Statistical analysis

Categorical variables were described using counts and percentages. Continuous variables were expressed as the mean and standard deviation or median and interquartile range, depending on the result of the Kolmogorov-Smirnov test. To detect significant differences between causes of IA-related and IA-unrelated death we used the chi-square or Fisher’s exact test for categorical variables and the Student’s *t* test or Mann-Whitney *U* test for continuous variables, as appropriate. A multivariate analysis to determine independent risk factors for IA-related mortality was performed comparing patients with IA-related death versus all other patients. Variables shown to be significant in the univariate analysis and which were considered clinically important were entered into the multivariate analysis. The relative risks were expressed as hazard ratios (HR) and 95% confidence intervals (CI).

The results were analyzed using SPSS version 15.0 (SPSS Inc., Chicago, IL, USA). Statistical significance was set at α = 0.05. All reported p values are two-tailed.

## Results

### Study population and causes of death

A total of 152 patients with IA were enrolled in the study. Table 1 summarizes the characteristics of patients hospitalized for IA, as well as their clinical features, diagnosis, and treatment. Overall mortality (90 days) was 60.5% (92 of 152 patients). The median time to death after diagnosis was 16 days (IQR 6.25–33.5). The immediate causes of death are detailed in Table 2.

**Table 1:** Patient baseline characteristics, clinical features, diagnosis, and treatment.

Characteristics		Patients n = 152	%
Age, median years (IQR)		60 (49–67)	-
Male sex		93	61.2
Underlying disease			
	Hematologic malignancy	67	44.1
	Solid organ transplant	34	22.4
	Hematopoietic stem cell transplant	13	8.6
	Solid tumor	13	8.6
	AIDS	9	5.9
	Immunodeficiency disorder	5	3.3
	Other^&^	12	7.8
Immunologic risk^#^			
	Neutropenia	49	32.2
	Corticosteroid therapy	89	58.6
	Any immunosuppressive therapy	99	65.1
Infection site			
	Pulmonary only	136	89.5
	Disseminated IA	16	10.5
Diagnosis^#^			
	Culture^$^	113	74.3
	Galactomannan	95	62.5
	Biopsy or autopsy	34	22.4
Type of IA			
	Proven	38	25.0
	Probable	114	75.0
Primary antifungal therapy*			
	Voriconazole monotherapy	61	40.1
	Voriconazole-containing regimen	92	60.5
	Amphotericin B monotherapy^@^	19	16.8
	Combination therapy	25	16.4

^&^ Contains patients with severe immunosuppressive treatment, mainly high dose of corticosteroids.

^#^ Patients could have >1 characteristics within a category.

^$^ *A*. *fumigatus*, 87 cases (76.9%); *A*. *niger*, 6 (5.3%); *A*. *terreus*, 6 (5.3%); *A*. *flavus*, 12 (10.6%) other, 6 (5.3%).

* Systemic antifungal therapy with anti-Aspergillus activity given for at least 5 consecutive days.

^@^ Liposomal amphotericin B 13 (11.5%); Lipidic amphotericin B 6 (5.3%).

**Table 2:** Immediate cause of death for patients with IA.

Cause of death^1^		IA-related death N = 62 (%)	IA-unrelated death N = 30 (%)	Total N = 92 (%)	p^2^
Respiratory failure		50 (80.6)	9 (30)	59 (64.1)	<.001
	- caused primarily by *Aspergillus* infection	24 (38.7)	0	24 (26.1)	<.001
	- caused by *Aspergillus* infection and concomitant lung infections^3^	14 (22.6)	6 (20)	20 (21.7)	.99
	- caused by *Aspergillus* infection and severe comorbidities^4^	12 (19.4)	3 (10)	15 (16.3)	.37
Underlying disease^5^		6 (9.7)	12 (40)	18 (19.6)	<.01
Septic shock caused by bloodstream infection^6^		5 (8.1)	11 (36.7)	14 (15.2)	<.01
Pulmonary hemorrhage^7^		10 (16.1)	0	10 (10.9)	.03
Neurological conditions^8^		7 (11.3)	1 (3.3)	8 (8.7)	.27
Multiorganic failure^9^		7 (11.3)	0	7 (7.6)	.09
Other^10^		2 (3.2)	12 (40)	14 (15.2)	<.001

^1^ More than >1 cause of death was considered in 38 patients.

^2^ Differences between IA-related and IA-related mortality.

^3^ Cytomegalovirus, 7 patients; *Pseudomonas aeruginosa*, 5 patients; *Pneumocystis jirovecii*, 3 patients; Influenza A(H1N1)pdm09, 3 patients; *Streptococcus pneumoniae*, 2 patients; nocardiosis, 2 patients; *Rhodococcus equi*, 1 patient; respiratory syncytial virus, 1 patient; aspiration pneumonia, 1 patient. Three respiratory co-pathogens were found in 5 patients.

^4^ Severe chronic obstructive pulmonary disease, 8 patients; GVHD, 3 patients; and in 1 patient each: cerebrovascular disease, lung cancer, pulmonary fibrosis, and acute pulmonary thromboembolism.

^5^ Acute myeloid leukemia relapse, 6 patients; graft failure in organ solid recipients, 4 patients (two lung, one kidney, one liver); GVHD, 4 patients with allo-hematopoietic stem cell transplantation; cavum massive hemorrhage secondary to solid cancer, 1 patient; advanced lung cancer, 1 patient; intestinal obstruction in patient with metastatic cancer, 1 patient; severe aplasia after chemotherapy in one patient with chronic lymphocytic leukemia.

^6^ Gram-negative bacilli, 6 patients; *Enterococcus spp*., 4 patients; *Listeria monocytogenes*, 1 patient; *Streptococcus pneumoniae*, 1 patient; Candida albicans, 1 patient; polymicrobial bacteremia with *Enterococcus faecim* and *Achromobacter dentrificans*, 1 patient.

^7^ Necrotizing pneumonia caused by *Aspergillus* in patients with severe pancytopenia due to hematologic disease, 5 patients (in one case co-infection with *Pseudomonas aeruginosa* was found); necrotizing pneumonia caused by *Aspergillus* in patients with pancytopenia and/or coagulopathy due to liver disease, 3 patients; necrotizing pneumonia caused by *Aspergillus*, 1 case; necrotizing pneumonia caused by *Aspergillus* and *Pseudomonas aeruginosa*, 1 case.

^8^ Brain herniation caused by focal lesion +/- cerebral hemorrhage caused by *Aspergillus* in central nervous system, 5 patients; cerebral ischemic event, 2 patients; primary brain hemorrhage, 1 patient.

^9^ Disseminated invasive aspergillosis with multiorganic failure, 3 cases; multiorganic failure caused by respiratory failure due to *Aspergillus* and liver failure due to severe underlying liver disease, 3 patients; multiorganic failure caused by respiratory failure due to *Aspergillus* and heart failure after cardiac transplantation, 1 patient.

^10^ *Clostridium difficile* infection, 4 patients (co-infection with CMV was found in 1 case); sudden cardiopulmonary arrest in patients with multifactorial encephalopathy, 2 patients; sudden cardiac arrest, 2 patients; acute myocardial ischemia, 1 patient; neutropenic colitis, 1 patient, intestinal ischemia and secondary peritonitis, 1 case; post-surgical esophageal perforation and secondary mediastinitis, 1 case; severe cachexia (adult/32 kg), 1 case; diabetic ketoacidosis, 1 case.

Mortality was judged to be IA-related in 62 cases (67.4% of deaths; 40.8% of patients). Of these, IA was the cause of death in 36 cases (39.1% of deaths; 23.7% of patients) and was judged to have played a major role in the patient’s death in the remaining 26 cases (28.3% of deaths; 17.1% of patients). Mortality was defined as IA-unrelated in 30 cases (32.6% of deaths; 19.7% of patients): IA played a minor role in 18 cases (19.5% of deaths; 11.8% of patients) and had no role in the patient’s death in 12 cases (13.0% of deaths; 7.9% of patients).

Table 2 shows the differences in cause of death between patients with IA-related versus IA-unrelated mortality. The most common cause of IA-related death was respiratory failure (50 of 62 patients; 80.6%), caused primarily by *Aspergillus* infection, although also by concomitant infections or severe lung comorbidities. Progression of underlying disease and septic shock caused by bloodstream infection were the most frequent causes of IA-unrelated death.

Fifteen patients were diagnosed after death. Autopsyprovided the diagnosis of five patients in whom the diagnostic tests to rule out IA were not performed during hospitalization. Positive cultures and/or galactomannan results reported after death, plus necropsy results, allowed a diagnosis to be made in a further five patients, while positive cultures and/or galactomannan results reported after death enabled the remaining five patients to be diagnosed. Disseminated IA was documented in 6 of these 15 patients (40%). IA was judged to have been the cause or to have played a major role in the patient’s death in all 15 of these cases.

### Timing of IA-related and IA-unrelated mortality

**Fig 1:**
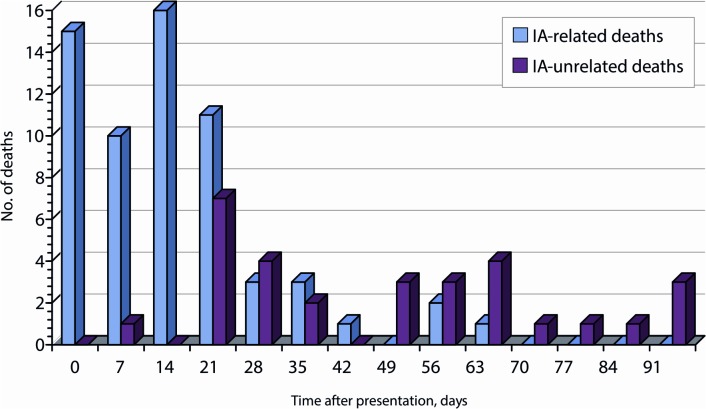
Frequency plot of IA-related and IA-unrelated mortality.

Survival plots and frequency distributions of death by time for IA-related and IA-unrelated mortality are shown in Fig. 1. There were significantly different patterns in time to death for patients with IA-related versus IA-unrelated mortality. Median survival days for patients with IA-related death was 10 (IQR 1.5–18.25), as compared with 43.5 (IQR 18.75–60.25) for patients with IA-unrelated death (p<.001).

IA-related mortality accounted for 97.6% of deaths within the first 14 days and 86.7% of deaths within the first 21 days. IA-unrelated mortality accounted for 58% of deaths from day 14 to day 90 and 68.6% of deaths from day 21 to day 90 (p<.001 for both comparisons). Of the IA-related deaths, 66.1% occurred within 14 days, and 83.9% within 21 days. No IA-related death was documented after 60 days of follow-up. The odds of an IA-related death occurring within 14 and 21 days of presentation were 19.84-fold and 3.15-fold higher than that of an IA-unrelated death, respectively.

### Factors associated with IA-related mortality

Table 3 shows the independent factors associated with IA-related mortality. After adjustment, chronic liver disease (HR 4.542; 95% CI, 1.69–12.23) was the only factor independently associated with IA-related mortality. Conversely, receipt of voriconazole was independently associated with reduced risk of IA-related death (HR 0.43; 95% CI, 0.20–0.93).

**Table 3:** Independent risk factors for IA-related death.

Variable	Adjusted	
	HR (95% CI)	p
Patient-related factors		
Chronic liver disease	4.54 (1.69–12.22)	.003
Severe impairment on PFT^1^	2.46 (0.90–6.77)	.081
Hematologic disease	0.99 (0.42–2.35)	.992
Corticosteroid treatment	1.37 (0.61–3.06)	.449
IA-related factors		
Disseminated IA	2.12 (0.58–7.69)	.253
Proven IA	2.23 (0.90–5.56)	.986
Voriconazole treatment^2^	0.04 (0.20–0.93)	.032

^1^Severe pulmonary function test abnormality.

^2^ Voriconazole received for at least 5 days.

## Discussion

This multicenter study describes contemporary causes of death for adults with IA. Our cohort is representative of patients with IA and has similar patient characteristics, clinical presentation, and antifungal therapy to those reported by the PATH Alliance [11], the largest recent prospective description of IA patients. Previous information regarding the processes leading to death in adults with IA is scarce. In the present study, overall mortality for patients with IA at 90 days was 60.5%. This mortality rate is high, probably due to the heterogeneous population. IA in non-neutropenic, non-cancer patients still has a very poor prognosis, contrasting to the increasingly better survival in hematologic patients, likely due to strategies of early diagnosis allowing early appropriate treatment. Our mortality rate finding concurs with that of other investigators who reported rates ranging from 35.6% to 66% [5–8, 12]. Recently, Marr et al. [13] reported a mortality rate at 6-weeks close to 30% in patients who had an underlying hematologic malignance or hematopoietic cell trasplantation diagnosed with possible, probable, or proven IA. Mortality was judged to be IA-related in 62 of 152 cases (40.8%), accounting for 67.4% of deaths; in the remaining 32.6% of cases death was due to IA-unrelated causes. These data are difficult to compare with those obtained by other researchers, owing to differences in definitions and study populations. Importantly, we found substantial differences in causes and timing for IA-related and IA-unrelated mortality.

In our study the most frequent cause of IA-related death was respiratory failure. This was mostly a direct result of *Aspergillus* infection, although there were also cases of respiratory failure in patients with coexisting infections or severe comorbidities. Pulmonary hemorrhage was another frequent cause of IA-related death. These findings suggest the need for measures to improve the care of pulmonary function in the early management of IA. It is important to note, however, that IA is not a homogeneous disease. Its pathogenesis differs depending on the host’s immune status [14–17], and the lung injury could occur through different pathways. Early rollout and treatment of coinfections, as well as optimized management of chronic pulmonary diseases, would also seem mandatory.

In our study the progression of underlying disease and septic shock caused by bacteremia were the most frequent causes of IA-unrelated death. The prognosis of patients beyond 21 days of IA diagnosis could be greatly influenced by improving the treatment of underlying diseases and/or preventing and managing bacterial infections.

Although our cohort of patients was recruited at a time of improved diagnostic tests such as chest CT and the galactomannan antigenemia assay, some cases of IA were still only diagnosed at autopsy or post-mortem. In line with previous reports [5] these patients were more likely to have disseminated disease. These results show that more prompt diagnosis of IA remains a challenge for physicians.

Most of the IA-related deaths (83.9%) occurred within the first 21 days after diagnosis. In fact, IA-related mortality accounted for 97.6% of deaths within the first 14 days. After this period the number of IA-related deaths diminished rapidly, with no IA-related death being documented after 60 days. By contrast, IA-unrelated mortality accounted for 68.6% of deaths from day 21 to day 90 after IA diagnosis. These results suggest that assessment of survival at 12 weeks after diagnosis (a criterion used by many researchers) [5, 6, 12] is an imprecise indicator of the efficacy of IA treatment. This finding concurs with that of other investigators [18]. Given that the 12-week period includes a large number of deaths due to competing causes that are not related to fungal infections, researchers interested in assessing treatment response in IA infection should therefore focus only on IA-related deaths. We believe that our study used clear and uniform definitions that were intended to be reproducible in future studies. Studies not using an independent clinical expert review committee to determine the cause of death for patients should apply a cut-off of 14 days so as to provide relevant information only about deaths directly caused by IA. Twenty-one days (3 weeks) seems to be a good cut-off for a global evaluation of IA-related mortality. However, our data are retrospective and subject to limitations. Further prospective studies to assess the optimal cutt-off point to evaluate treatment response in IA infection should be conducted.

We found that the presence of liver disease was independently associated with an increased risk for IA-related mortality. The association between hepatic impairment and increased risk for mortality in patients with IA has been previously documented in hematopoietic cell transplant recipients [5].Importantly, we found that voriconazole treatment was independently associated with reduced risk of IA-related death. The better mortality outcomes of patients treated with voriconazole compared with amphotericin B therapy for IA has been demonstrated in observational studies [5, 6] and in a randomized trial [12]. Our study has several limitations. One potential weakness is that the validity of using a clinical review committee to determine the cause of death for patients with IA has not been previously established. Nevertheless, this method was chosen because it was the most practical in nature and is likely to provide more reliable data than would death certificates, most of which are signed by inexpert practitioners with limited experience in the management of these complicated patients. Similar clinical consensus methods have been used to classify mortality for many other conditions [19,20]. It is nonetheless difficult to avoid subjective points of view when establishing causes of death in these patients. We sought to address this by using precise definitions of IA-related and unrelated mortality that were applied in a uniform manner. Researchers were blind to the timing of mortality when determining the cause of death, and all decisions were agreed by consensus. A final limitation to consider is that an autopsy could not be performed for all the patients who died.

In conclusion, this study describes the immediate causes of death in a current cohort of patients with IA. The findings suggest that better management of lung injury within the first 21 days after IA diagnosis is the main challenge for physicians in terms of improving IA outcomes. Importantly, there were significant differences in causes and timing between IA-related and IA-unrelated mortality and these should be considered in future research in order to assess the quality of IA care.
